# A CASE OF PAINFUL CENTROFACIAL NODULE WITH DISCHARGING SINUSES AND BLACKISH NASAL DISCHARGE

**DOI:** 10.4103/0019-5154.53172

**Published:** 2009

**Authors:** Saumya Panda

**Affiliations:** *From the Department of Dermatology, KPC Medical College, Kolkata, India*

A 28-year-old male presented with a swelling on the left side of the nose extending up to the left side of the forehead and encroaching the left side of the upper lip and angle of the mouth. The swelling appeared unobtrusively by the side of his nose and increased in size gradually. Lately, over the last month or so, the swelling became increasingly painful. Characteristically, the pain appeared to increase at night and disturbed his sleep. Since the past 2 weeks, a blackish, granular discharge had started from his left nostril and a couple of sinus openings on the left paranasal area. There was no discharge from the other side. Like the pain, the discharge too had a tendency to worsen whenever the patient laid down.

Among other symptoms, a constant headache had begun. There was also chronic sleeplessness and anxiety, which could be partly ascribed to the pain and nasal discharge that were aggravated by the supine position. The patient also claimed to have lost some weight, but was unsure about the actual magnitude of the weight loss.

The patient first visited an otolaryngologist who treated him with some antibiotics that had no effect. Later, he visited a couple of dermatologists, both of whom advised a biopsy, which he declined. He then turned to a homeopath and it was his advice that he was following for the last couple of months. As things gradually worsened, the patient decided to seek our professional advice.

There was no significant history of any concurrent illness. The patient was accompanied by two older brothers during the consultation, who denied that there was similar illness, either currently or in the past, among any other members of the family. The man lived as part of a large joint family and was self-employed running a telephone booth at his home.

Upon examination, a moderately tender, ill-defined, very firm, erythematous, fixed solid nodule with sinuses discharging blackish granular substance was seen [Figure 1]. A blackish, thick mucopurulent discharge with necrotic debris was observed to be coming out of the left nostril [Figure 2]. A few soft, non tender cervical lymph nodes were palpable. Other than mild anemia, there was no other significant finding on systemic examination.

**Figure 1:**
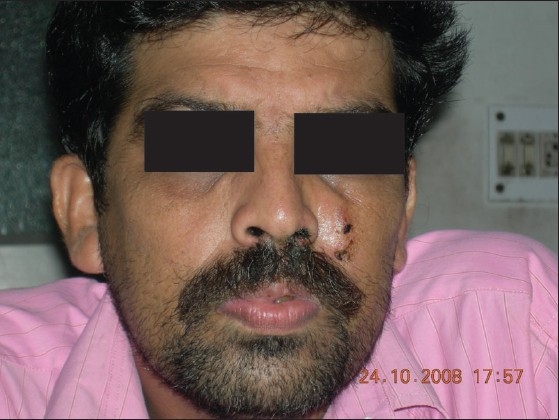
Swelling on the left side of the nose extending up to the forehead and including multiple sinuses

**Figure 2:**
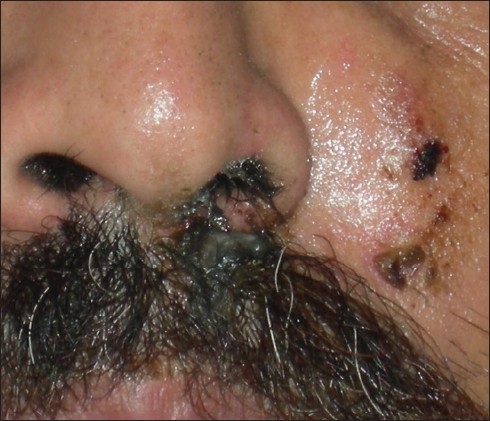
Close-up view of the sinuses and the blackish discharge from the left nostril containing purulent necrotic debris

On routine laboratory investigations, his hemoglobin level was 10.2%; total leucocyte count was 7,600/cubic mm; and the differential count was neutrophils 62%, lymphocytes 29%, monocytes 5%, and eosinophils 4%; the platelet count was 1,52,000/cmm. The erythrocyte sedimentation rate was 45 mm and C-reactive protein was 13. The liver function test was within normal limits. A venereal diseases reference laboratory (VDRL) test was negative. Serum creatinine was 0.8%. No abnormality was detected on a routine urine examination. The anti-nuclear autoantibodies, including the anti-double stranded-DNA antibody, were negative.

A full thickness skin biopsy was performed. The hallmark on hematoxylin-eosin staining was the presence of sheets and whorls of atypical spindle-shaped cells interspersed with a few giant cells and dyskeratotic cells [Figures 3 and 4]. The accompanying fibrosis was also prominent and seemed intercalating at several places. Continuity with the epidermis was noted in parts. On higher magnification, the spindle cells were seen to have a large vesicular nucleus and scanty eosinophilic cytoplasm, some of them with indistinct cell borders [Figure 5]. There was complete absence of any granulomas or fungal elements. Tissue from the lesional skin was sent together with the biopsy specimen for fungal culture, which showed the presence of *Blastomyces spp*.

**Figure 3:**
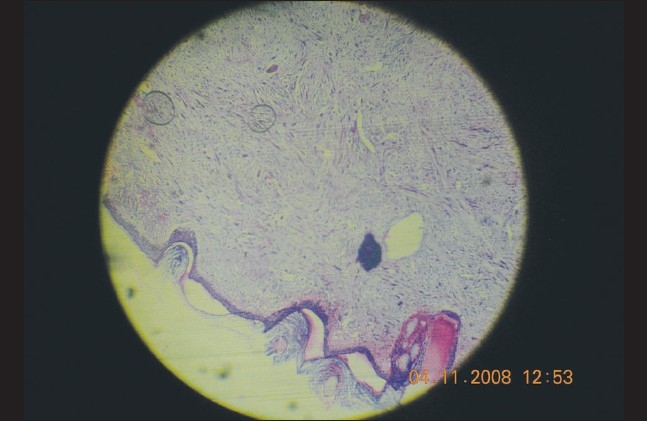
Whorls of atypical spindle cells extending at places from the epidermis (H&E, magnification ×100)

**Figure 4:**
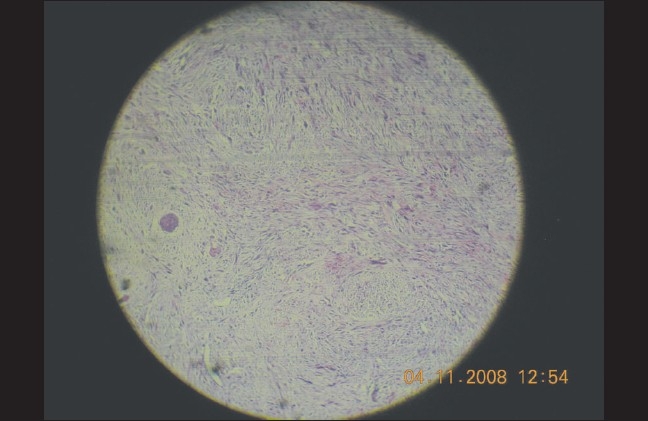
Sheets of atypical spindle cells with scattered giant cells (H&E, magnification ×100)

**Figure 5:**
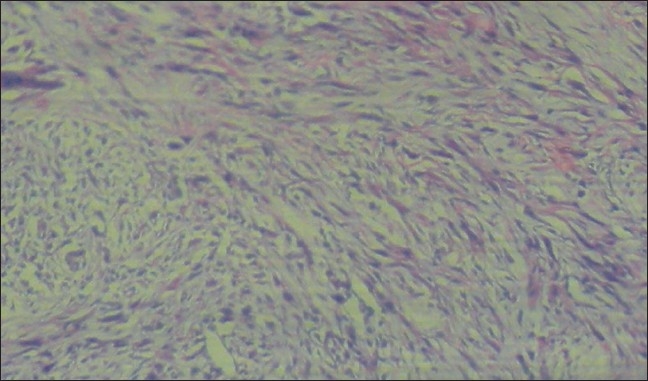
Atypical spindle cells with vacuolar, eosinophilic cytoplasm (H&E, magnification ×400)

On the basis of these reports, a computed tomography (CT) scan of the head and neck was ordered. At the same time, the patient was put on tab. itraconazole (200 mg twice daily) on the basis of the fungal culture report. The CT scan showed a left-sided paranasal mass encroaching upon the walls of the nasal cavity and the left maxillary sinus running up to a depth of 4 mm. No other space-occupying lesion was found. It was decided to submit the biopsy tissue for immunohistochemistry.

Staining for S-100, desmin, HMB-45, and CD-10 were all negative. Only cytokeratins (CK 5/6) were positively stained in a diffuse manner [Figure 6]. By the time these results were received the patient showed significant improvement on oral itraconazole within a period of 2 weeks [Figure 7].

**Figure 6:**
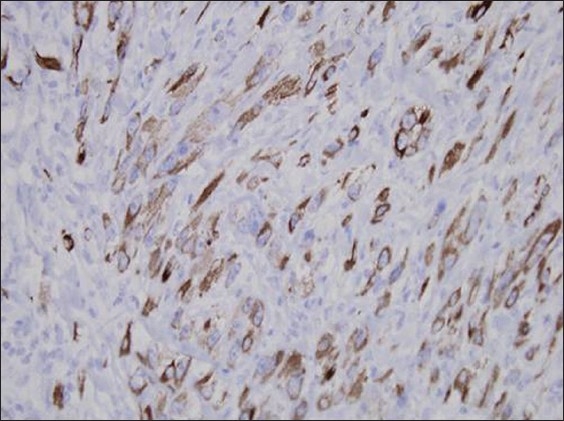
CK 5/6 stained cells (magnification ×40)

**Figure 7:**
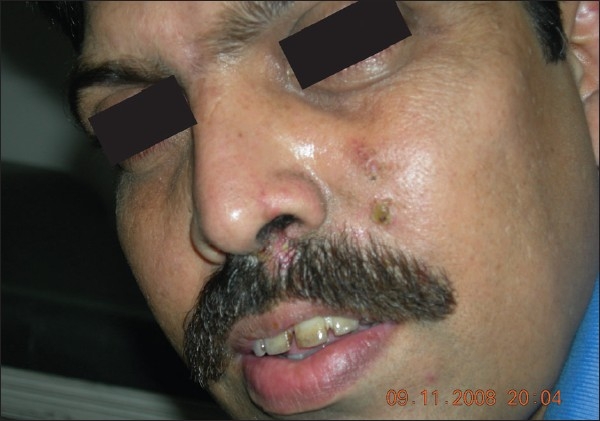
Significantly improved appearance with the absence of nasal discharge and dried-up sinuses following 2 weeks of itraconzole therapy

## Questions

1. What is the final diagnosis?
2. What could be the differential diagnosis following clinical examination?
3. What could be the probable diagnosis following the routine histopathology?

## Answers

1. The final diagnosis was sarcomatoid squamous cell carcinoma. Sarcomatoid or the spindle-cell variant of squamous cell carcinoma is a rare variant that usually arises in sun-damaged or irradiated skin.[1] It generally appears as an exophytic tumor or an ulcerated mass on the sun-exposed skin of elderly patients.[2] The case under discussion is an atypical presentation of this rare entity as it has presented in an immunocompetent, non-irradiated young adult in the third decade without any external ulceration. Histologically, it is composed of atypical spindle cells arranged in a whorled pattern. Unlike conventional squamous cell carcinoma (SCC), the tumor cells infiltrate the dermis singly without the formation of nests or cords. The connection to the overlying epidermis, if present, is supporting evidence for the diagnosis. Mitoses and bizarre pleomorphic giant cells may be frequently seen. Deep infiltration of the dermis, subcutis, and underlying fascia is common.
  Sarcomatoid SCC was initially reported by Martin and Stewart in 1935.[3] It was believed that previous radiation was the most important cause, as 6 out of the 8 patients reported had a history of radiation. It was also thought to be an aggressive form of SCC, as 4 of these 8 patients died of cancer. This was disputed in 1950 in a report of five cases by Strauss in which none of the patients had a history of radiation exposure.[4] In 1972, Smith proposed that when spindle cell SCCs arise in a site of previous radiation, they tend to have a more aggressive course, as would be expected. When they arise de novo, Smith proposed that these lesions do not exhibit a more aggressive behavior than conventional SCC.[5] Spindle cell SCC has also been reported in renal transplant recipients, in which 1 of 4 patients developed metastatic disease.[6] Unfortunately, no large studies have been conducted regarding the prognosis of spindle cell SCC, especially comparing de novo lesions with radiation-associated lesions.
2. The clinical morphology gave rise to several possibilities. Among them are subcutaneous fungal infections, rhinoscleroma, mycobacterial infections, cervicofacial actinomycosis, Wegener's granulomatosis, lethal midline granuloma, syphilis, Churg-Strauss syndrome, sinonasal sarcoidosis, fibrocytic tumors, such as atypical fibroxanthoma, malignant fibrous histiocytoma, squamous cell carcinoma, basal cell carcinoma, desmoplastic melanoma, polymorphic reticulosis, berylliosis, etc.
3. On routine histopathology, all the granulomatous or infective etiologies could be effectively ruled out. Despite the positive report of fungal culture, blastomycosis was an unlikely possibility on clinical grounds (absence of warty growths, etc.). The absence of either fungal elements or granulomas helped to discount the possibility altogether. The positive fungal culture and the improvement on oral itraconozole could be ascribed to secondary infection.
  The differential diagnosis could be narrowed down to spindle cell tumors, such as atypical fibroxanthoma including its non-pleomorphic variant,[7] spindle cell melanoma, soft tissue tumors, and spindle-celled SCC. This was one of those cases where immunohistochemistry was imperative in order to diagnose and to prognosticate. Spindle cell and desmoplastic melanoma and poorly differentiated cutaneous metastases could be negated by the S-100 protein negativity. Soft tissue tumors were negated by the absence of the intermediate filament desmin. The non-staining by HMB-45 further bolstered the absence of involvement of melanocytes. The presence of high molecular weight cytokeratins (CK5/6) and the absence of CD10 helped the diagnosis in favor of SCC as opposed to atypical fibroxanthoma, including the non-pleomorphic variant of the latter.[8]
  It may be remarked here that atypical fibroxanthoma, first described by Helwig in 1961,[9] is a spindle cell tumor of mesenchymal origin, that also presents (like spindle cell SCC) in the sun-damaged skin of the elderly and is morphologically indistinguishable from the latter on routine hematoxylin and eosin (H and E) stained sections. The non-pleomorphic variant of atypical fibroxanthoma, first described by Calonje *et al.* in 1993,[10] is so very indistinguishable from this variant of SCC as to be termed by some to be the same condition. In some of these cases, even immunohistochemistry is also not enough to differentiate between these conditions. For example, some poorly differentiated sarcomatoid SCCs may show loss of cytokeratin expression making the diagnosis even more challenging. In these cases, electron microscopy is the only recourse. The presence of tonofilaments and desmosomes confirms an epithelial origin.[11] Poorly differentiated sarcomatoid SCC, however, may not always have evidence of tonofilaments and desmosomes, making them indistinguishable from sarcomas.
